# Tn Antigen Expression Contributes to an Immune Suppressive Microenvironment and Drives Tumor Growth in Colorectal Cancer

**DOI:** 10.3389/fonc.2020.01622

**Published:** 2020-08-18

**Authors:** Lenneke A. M. Cornelissen, Athanasios Blanas, Anouk Zaal, Joost C. van der Horst, Laura J. W. Kruijssen, Tom O’Toole, Yvette van Kooyk, Sandra J. van Vliet

**Affiliations:** Department of Molecular Cell Biology and Immunology, Cancer Center Amsterdam, Amsterdam Infection & Immunity Institute, Amsterdam UMC, Vrije Universiteit Amsterdam, Amsterdam, Netherlands

**Keywords:** colorectal cancer, *O*-glycosylation, Tn antigen, anti-tumor immunity, tumor growth

## Abstract

Expression of the tumor-associated glycan Tn antigen (αGalNAc-Ser/Thr) has been correlated to poor prognosis and metastasis in multiple cancer types. However, the exact mechanisms exerted by Tn antigen to support tumor growth are still lacking. One emerging hallmark of cancer is evasion of immune destruction. Although tumor cells often exploit the glycosylation machinery to interact with the immune system, the contribution of Tn antigen to an immunosuppressive tumor microenvironment has scarcely been studied. Here, we explored how Tn antigen influences the tumor immune cell composition in a colorectal cancer (CRC) mouse model. CRISPR/Cas9-mediated knock out of the *C1galt1c1* gene resulted in elevated Tn antigen levels on the cell surface of the CRC cell line MC38 (MC38-Tn^high^). RNA sequencing and subsequent GO term enrichment analysis of our Tn^high^ glycovariant not only revealed differences in MAPK signaling and cell migration, but also in antigen processing and presentation as well as in cytotoxic T cell responses. Indeed, MC38-Tn^high^ tumors displayed increased tumor growth *in vivo*, which was correlated with an altered tumor immune cell infiltration, characterized by reduced levels of cytotoxic CD8^+^ T cells and enhanced accumulation of myeloid-derived suppressor cells. Interestingly, no systemic differences in T cell subsets were observed. Together, our data demonstrate for the first time that Tn antigen expression in the CRC tumor microenvironment affects the tumor-associated immune cell repertoire.

## Introduction

Tumor cells are frequently characterized by an aberrant glycosylation profile, hence, current research is focused on how tumor-associated glycan structures support tumor progression. Glycosylation is a post-translational modification of proteins and lipids, which is not template-driven, but instead, is influenced by the metabolic state of the cell and the availability of the sugar donors. Glycan structures are known to drive diverse biological functions, among others, protein folding, cell-cell and cell-matrix adhesion and cell signaling (1). Moreover, the ability of glycan structures to modulate immune responses has led to the hypothesis that tumor-associated glycan structures are responsible for skewing the tumor microenvironment toward an immune suppressive phenotype (2). However, our knowledge on how individual tumor-associated glycan structures actually affect anti-tumor immunity is still quite limited.

Compared to their non-malignant counterparts, tumor cells express much higher levels of the Tn antigen glycan structure (3, 4). Expression of Tn antigen is a prognostic factor for overall and relapse-free survival in lung adenocarcinoma (5) and correlates with metastatic potential in colorectal cancer (6). Tn antigen is made up of one *N*-acetylgalactosamine (GalNAc) monosaccharide and represents the initiation of mucin-type *O*-glycosylation. In humans, a repertoire of twenty GalNAc-transferases (GalNAcTs) is responsible for the initiation of *O*-glycosylation (7) and thus Tn antigen synthesis. The expression of GalNAcTs is dynamic and these enzymes have been shown to relocate from the Golgi to the ER in certain tumor types (8). Although contradictory findings have been published (9), this relocation is thought to enable a prolonged mode of action, leading to overexpression of Tn antigen, thereby promoting cancer cell invasiveness. Since epithelial cells produce high levels of mucin proteins that are heavily *O*-glycosylated, high expression of Tn antigen predominantly occurs in epithelial cancer types, including colorectal cancer (CRC), where 86% of primary and metastatic human CRC tissues express the Tn epitope (10).

Tn antigen overexpression has been shown to directly induce oncogenic features including enhanced cell proliferation, decreased apoptosis, increased adhesion and migratory capacities (11–13). Since immune cells highly express receptors recognizing glycan structures (14), inhibition of anti-tumor immunity might be an additional mechanism by which Tn antigen supports tumor growth. However, whether cancer cells exploit Tn antigen *in vivo* to evade immune attack has never been thoroughly investigated.

In the present study we assessed the impact of Tn antigen on *in vivo* tumor growth and the immune cell composition present at the tumor site using CRISPR/Cas9 glyco-engineered mouse colorectal cancer MC38 cells. We report that overexpression of Tn antigen drives tumor growth in CRC, which coincided with reduced tumor immune cell infiltration, increased myeloid-derived suppressor cells and decreased CD8^+^ T cell infiltration. Together, these data suggest that Tn antigen may promote an immune suppressive tumor microenvironment, which could contribute to tumor immune evasion and thus tumor progression.

## Materials and Methods

### CRISPR/Cas9 Constructs

CRISPR/Cas9 constructs were made using the pSpCas9(BB)-2A-Puro plasmid, a gift from Feng Zhang (Addgene #62988), according the previously described protocol (15). gRNA sequences for murine *C1galt1c1* were as follows: top strand CACCGGTTTTCTTACCTCCAAA; bottom strand CCAAAAGAATGGAGGTTTCAAA. The gRNA encoding plasmid was used for transformation of XL1-Blue Sublconing-Grade competent bacteria (Stratagene). Nucleobond Xtra Midi kit (Macherey-Nagel) was used to purify the plasmid according manufacturer’s protocol.

### Generation of the MC38-Tn^high^ Cell Line

MC38 cells were cultured in DMEM supplemented with 10% heat inactivated fetal calf serum (FCS, Biowest), 1% penicillin and 1% streptomycin. MC38 cells were transfected with CRISPR/Cas9 constructs either targeting the *C1galt1c1* gene (MC38-Tn^high^) or an empty CRISPR/Cas9 construct (MC38-MOCK). For transfection, Lipofectamine LTX with PLUS^TM^ reagent (ThermoFisher Scientific) was used and applied according to the manufacturer’s protocol. Transfected MC38 cells were selected in bulk based on their Tn antigen cell surface profile as described below. Transfected MC38 cells were incubated with 5 μg/mL of the biotinylated α-*N*-acetylgalactosamine-specific lectin *Helix Pomotia* agglutinin (HPA, Sigma) for 1 h on ice, washed with medium and subsequently incubated with streptavidin-PE (Jackson ImmunoResearch) again for 1 h on ice. Cells were washed with medium and sorted in bulk on HPA high binding cells. The sorting procedure was performed twice to obtain the final MC38-Tn^high^ cell line.

### Surveyor Assay

To obtain genomic DNA, fresh cells were harvested and DNA was isolated with the Quick-DNA^TM^ kit (Zymo research) according to manufacturer’s instructions. The *C1galt1c1* gene was amplified with qPCR (forward primer: CTGGCGGTCTGCCTGAAATA, reverse primer: TGTACAAGCAGACTTCAATG). The qPCR products (416 bp) were hybridized and treated with Surveyor Nuclease (Surveyor Mutation Detection Kit, Integrated DNA Technologies), which recognizes and cleaves any DNA mismatches. To visualize the mutation, the Surveyor Nuclease-treated products were separated by DNA agarose gel electrophoresis.

### T Synthase Assay

Cell lysates were obtained using 0.5% Triton X-100 in TSM (20 mM Tris-HCl, pH 7.4, 150 mM NaCl, 2 mM MgCl_2_, 1 mM CaCl_2_) and subsequently used for the T synthase assay as described by Ju and Cummings (16). Shortly, T synthase present in cell lysates utilizes the commercially available acceptor-substrate GalNAcα-4MU (Sigma Aldrich) and the donor-substrate UDP-Gal (Sigma Aldrich) to generate Galβ1-3GalNAcα-4MU structure. This product is hydrolyzed by *O*-glycosidase (New England Biolabs), leading to free 4-MU that is highly fluorescent and can be measured at an excitation of 355 nm and emission of 460 nm. The protein concentration in the cell lysate was determined with Pierce^TM^ BCA protein assay kit (ThermoFisher Scientific) to determine T synthase enzyme activity per microgram protein.

### Determination of the Cellular Glycosylation Profile

MC38 cells were incubated with 5 μg/mL of the HPA or peanut agglutinin (PNA, Vector Laboratories) lectin, anti-Tn antigen antibodies [5F4, kindly provided by H. Clausen and H. Wandall (17)] or 10 μg/mL of mouse MGL-2-Fc for 30 min at 37°C. Cells were washed and incubated with streptavidin-APC (BD Biosciences), goat anti-mouse IgM-A488 (ThermoFisher Scientific) or goat anti-human IgG-FITC (Jackson ImmunoResearch) respectively for 30 min at 37°C. Cells were washed and acquired on the Beckman Coulter Cyan flow cytometer. Data was analyzed with FlowJo v10.

### CellTiter-Blue^®^ Cell Viability Assay

The CellTiter-Blue^®^ Cell Viability assay (Promega) was used according to the manufacture’s protocol to measure the metabolic activity of MC38 cells. Briefly, 30.000 MC38 cells were cultured in a 1:6 dilution of the CellTiter-Blue^®^ Reagent. The metabolic activity was measured during the first 24 h of culture using a FLUOstar Galaxy (MTX Lab systems) with an excitation and emission of 560 and 590 nm, respectively.

### mRNA Library Preparation

The mRNA library was prepared as described previously (18). MC38 cell lines (passage 4-6) from three independent passages were harvested at three independent time points with 1% Trypsin EDTA and washed with PBS. Total RNA was extracted with a standard TRIzol isolation protocol (Thermo Fisher, 15596018). Quantity and purity were tested using the Nanodrop-2000 spectrophotometer (Nanodrop Technologies, United States). The library was synthesized using the TruSeq^®^ Stranded mRNA Sample preparation kit (Illumina, RS-122-9004), according to manufacturer’s LS protocol. The product quality during library generation was analyzed on the Agilent 2100 Bioanalyzer using the DNA 7500 chip (Agilent Technologies, 5067-1506).

### RNA-Sequencing, Alignment and Differential Expression Analysis

The library was sequenced on the HiSeq4000 instrument (Illumina) with a single read type of 50 bp (Tumor Genome Analysis Core, VUmc, Amsterdam, Netherlands) using standard Illumina protocols. RNA-sequencing reads were quality trimmed using Sickle (v1.33) (19) and quality checked using FASTQC^1^ (20). Reads were aligned to the Ensemble M. Musculus genome (build GRCm38.90) using HiSat2 (v2.0.4) (21) and subsequent processing was performed with samtools (v0.1.19) (22). FeatureCounts (R package Subread v1.5.0-p3)^2^ (23) was used to quantify aligned reads, excluding multi-overlapping reads.

The R package edgeR (v3.18.1) (24, 25) was used for library size adjustment, trimmed mean of M-values (TMM) normalization and differential expression analysis. Multidimensional scaling (MDS) plots were used to visualize sample distribution among MC38 cell lines. For differential expression analysis, the negative binomial dispersion was shrunken toward the common dispersion. EdgeR’s exact test for two-group comparison was used for computing *p*-values. Statistical differences in mRNA expression were identified using the following pairwise comparisons: (1) MC38-WT vs MC38-MOCK cells and (2) MC38- Tn^high^ vs MC38 MOCK cells. Here, per comparison, genes with more than 4 zeros across the 6 samples were discarded *a priori*. Significance was assessed using Benjamini-Hochberg false discovery rate (FDR) <0.05. Sequencing data is publicly available at the Sequence Read Archive (SRA) Gene Expression Omnibus thought GSO Series accession number GSE143700.

### Comparative Analysis of Gene Sets

Comparative analysis was performed among the differentially expressed genes (DEGs) in the (1) MC38-WT vs MC38-MOCK cells (1446 genes; Supplementary Table 1) MC38-Tn^high^ vs MC38 MOCK cells (2237 genes; Figure 2A and Supplementary Table 2) using Venny (v2.1.0)^3^. Of the 2237 DEGs in the MC38-Tn^high^ cells, only 1,343 genes were specifically affected in MC38-Tn^high^. The other 894 genes were also found in the comparison between MC38-WT and MC38-MOCK. Analyzing the direction of the log2 fold change of these 894 overlapping DEGs revealed that 5 of these DEGs showed a different direction of change between the two datasets (MC38-WT vs MC38-MOCK and MC38-Tn^high^ vs MC38-MOCK), meaning that these genes were actually differentially expressed in both datasets. Thus, analysis of the 894 overlapping genes yielded an additional 5 DEGs that were included for further analysis in the MC38-Tn^high^ set. Together, this resulted in 1348 DEGs in the MC38-Tn^high^ cells, of which the expression of 641 genes was suppressed and the expression of 707 genes was increased in MC38-Tn^high^ cells compared to MC38-MOCK cells (Figure 2B and Supplementary Tables 3A,B).

### Gene Ontology Term Enrichment and Pathway Analysis

Gene Ontology (GO) term enrichment analysis was performed on the 1348 DEGs in the MC38-Tn^high^ cells using Cytoscape v3.6.0^4^ (26), and the ClueGO plugin v2.5.0^5^ (27). Significantly enriched GO terms (Benjamini-Hochberg correction, False discovery rate (FDR) <0.05) were determined with the ontology source GO_BiologicalProcess-EBI-Quick-GO-GOA, and were subsequently visualized using view style Groups, GO level 6–13, and a kappa score threshold of 0.4 (Supplementary Table 4). Normalized counts of GO term-associated genes were visualized in heatmaps using Morpheus^6^. Genes assigned to GO terms on antigen presentation and T cell activation (GO level 3–8, FDR < 0.05, Supplementary Table 5) were included in the heatmap depicted in Figure 2D (*Bcl10*, *Erap1*, *Kcnn4*, *Pawr*, *Prkd2*, *Ptprj*, *Tap1*, *Tapbp*, *Tapbpl*, *Usp9x*). Hierarchically clustering was applied on cell line and log2 normalized counts for the DEGs using an one minus pearson correlation.

### *In vivo* Tumor Experiments

C57BL/6 mice were used at 8–12 week of age and bred at the animal facilities of Amsterdam UMC/VU University Medical Center (VUmc). An equal distribution of female and male mouse was used for all *in vivo* experiments. Experiments were performed in accordance with national and international guidelines and regulations. Each mouse received 2 × 10^5^ tumor cells in 100 μl PBS, which was subcutaneously injected in the flanks. Tumor measurements were performed three times per week in a double blinded manner. The total tumor volume was calculated according the formula 4/3 × π × *abc* (*a* = the radius of the width, *b* = the radius of the length and *c* = the average radius of width and length). Mice were sacrificed at day 13 or when the tumor reached a size of 2,000 mm^3^. The tumor, tumor draining lymph nodes and spleens were isolated and used for further experiments.

### Tumor and Organ Dissociation

First, tumors were finely minced and enzymatically digested for 25 min at 37°C in RPMI containing 1 mg/mL Collagenase type 4 (Worthington), 30 units/mL DNase I type II (Sigma-Aldrich) and 100 μg/mL hyaluronidase type V (Sigma-Aldrich). Lymph nodes and spleens were finely minced and digested for 10 min at 37°C with 2 U/mL Liberase TM (Roche) containing 30 units/mL DNase I type II. Subsequently, cell suspensions were passed through a 70 μm cell strainer and washed once with RPMI supplemented with 10% FCS, 1% penicillin, 1% streptomycin and 1% glutamax. Spleen digestions were subjected to ACK lysis and, together with the lymph node digestions, washed twice before use in subsequent experiments. To enrich for the lymphocytes and to remove dead cell debris from tumor digestions, the tumor digestion mixture was loaded on a Ficoll gradient. The interface was collected, washed twice and used for subsequent flow cytometric analysis.

### Flow Cytometric Analysis

To block Fc receptors, cells were pretreated with 2.4G2 (anti-CD16/32) for 10 min at RT. Cell viability was measured using a fixable viability dye (FVD, Zombie NIR, Biolegend). Cells were stained for 20 min at RT using the following cell surface markers: anti-CD45-PerCp (30-F11), anti-CD3-BV510 (17A2), anti-CD4-A700 (GK1.5), anti-CD8b-FITC (YTS156.7.7), anti-CD11b-BV605 (M1/70), anti-Gr-1-PE-Cy7 (RB6-8C5), anti-CD11c-BV785 (N418), anti-MHC-II-A700 (M5/114.15.2), anti-PD-1-BV785 (29F.1A12), anti-TIM-3-PE-Cy7 (RMT3-23) (all Biolegend). To stain for Foxp3, cells were fixed and permeabilized (Foxp3 Transcription Factor Staining buffer set, eBioscience) and incubated with anti-Foxp3-PE antibody (150D, Biolegend) for 20 min at RT. Additionally, tumor-infiltrating lymphocytes were restimulated *in vitro* with 100 ng/mL PMA and 500 ng/mL ionomycim and Brefeldin A was added to block cytokine secretion. After 5 h, cells were incubated with 2.4G2 and fixable viability dye (FVD, Zombie NIR) as described above and subsequently fixed and permeabilized using BD Cytofix/Cytoperm^TM^ (BD Biosciences) according to the manufacturer’s protocol and stained for 30 min at 4°C with anti-IFNγ-APC (eBiosciences). All samples were acquired on BD LSRFortessa^TM^ X-20. Data was analyzed with FlowJo v10.

### Statistical Analysis

Statistical significance was assessed using the GraphPad Prism software by performing an unpaired non-parametric *t*-test (^∗^*p* < 0.05, ^∗∗^*p* < 0.01, ^∗∗∗^*p* < 0.001; ns, non-significant).

## Results

### Loss of *C1galt1c1* Increases Tn Antigen Expression

**FIGURE 1:**
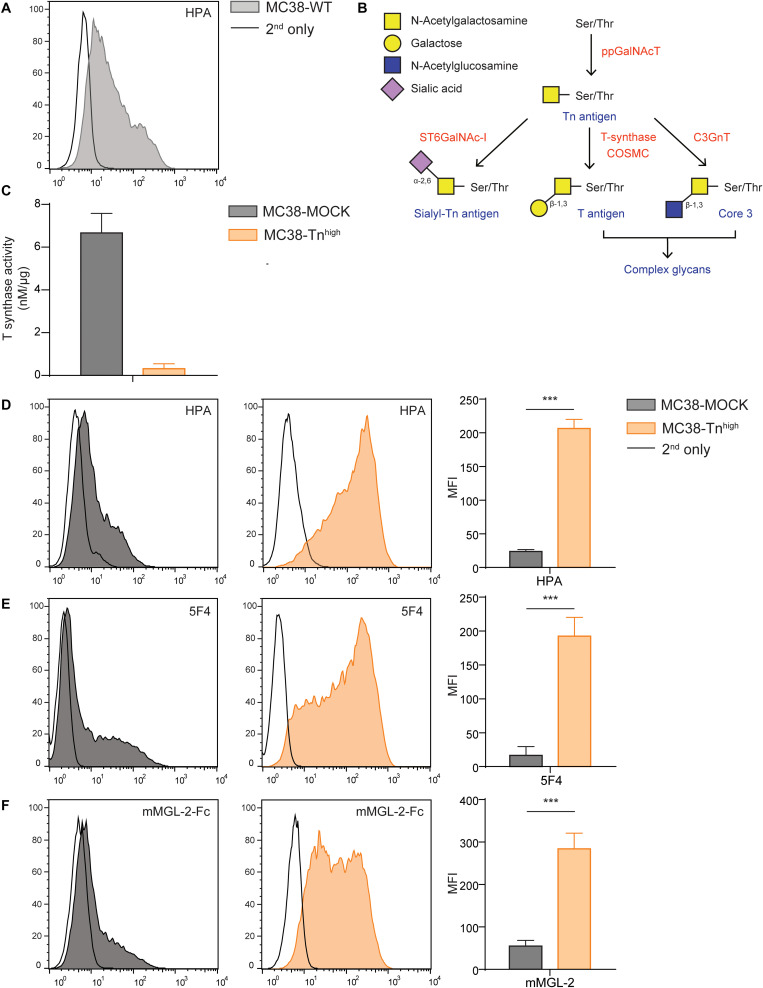
*C1galt1c1* knock out in MC38 cells results in high expression of Tn antigen. **(A)** Expression of Tn antigen on MC38 wild type (MC38-WT) cells analyzed by flow cytometry using the lectin HPA. **(B)** Simplified representation of the *O*-glycosylation pathway, adapted from Cornelissen and Van Vliet (29). **(C)** T synthase enzyme activity measured using the T synthase assay (16) in MC38 cells. **(D)** CRISPR/Cas9-mediated knock out of *C1galt1c1* resulted in increased Tn antigen expression (MC38-Tn^high^) as measured with HPA. As a negative control, MOCK-transfected cells (MC38-MOCK) were used. **(E,F)** Tn antigen expression measured with an anti-Tn antigen antibody (5F4) **(E)** or mouse macrophage galactose-type lectin (MGL) 2-Fc protein (mMGL-2-Fc) **(F)** on MC38 cells. Mean ± SD; MFI, mean fluorescent intensity, ***p < 0.001.

In this study we aimed to dissect the effect of high Tn antigen expression on colorectal cancer (CRC) tumor growth and the immune cell composition at the tumor site. Therefore, we first analyzed the steady state levels of Tn antigen on the mouse CRC cell line MC38 (MC38 wild type, MC38-WT), using the GalNAc-specific snail lectin *Helix Pomatia* agglutinin (HPA), and observed that our MC38-WT cells displayed only intermediate Tn antigen levels (Figure 1A). Normally, the T-synthase enzyme and its chaperone COSMC further elongate the Tn antigen with galactose, thus forming the T antigen (Galβ1-3GalNAcα-Ser/Thr) epitope (28) [see Figure 1B for a schematic representation of the *O*-glycosylation pathway, adapted from Cornelissen and Van Vliet (29)]. Indeed, CRISPR/Cas9-mediated knock out of the *Cosmc* gene (*C1galt1c1*) abolished T-synthase enzymatic activity (Figure 1C). Furthermore, *C1galt1c1* gene mutations were validated using the Surveyor assay (Supplementary Figure 1A). Consistently, Tn antigen levels were elevated after *C1galt1c1* gene knock out (MC38-Tn^high^), as confirmed by the increased staining of HPA (Figure 1D) and an anti-Tn antibody (5F4, Figure 1E), as well as the stronger binding of the mouse macrophage galactose-type lectin 2 (mMGL-2-Fc, Figure 1F), known for its specificity for the Tn antigen (30). Indeed, expression of T antigen, measured using the T antigen-recognizing lectin peanut agglutinin (PNA), was strongly reduced in the MC38-Tn^high^ cells (Supplementary Figure 1B). Moreover, mouse MGL-1 (mMGL-1-Fc), recognizing the Lewis X and A structures (30), only showed negligible binding to MC38-MOCK and MC38-Tn^high^ cells (Supplementary Figure 1C). Mutating the *C1galt1c1* gene did not alter the viability or proliferation of MC38-Tn^high^ cells (Supplementary Figure 1D). Thus, CRISPR/Cas9-mediated gene knock out of *C1galt1c1* results in elevated expression of the Tn antigen epitope in MC38 without affecting its intrinsic proliferative capacity.

### Knockout of *C1galt1c1* in Tumor Cells Is Associated With a Decrease in Antigen Presentation and Cytotoxic T Cell Activation

**FIGURE 2:**
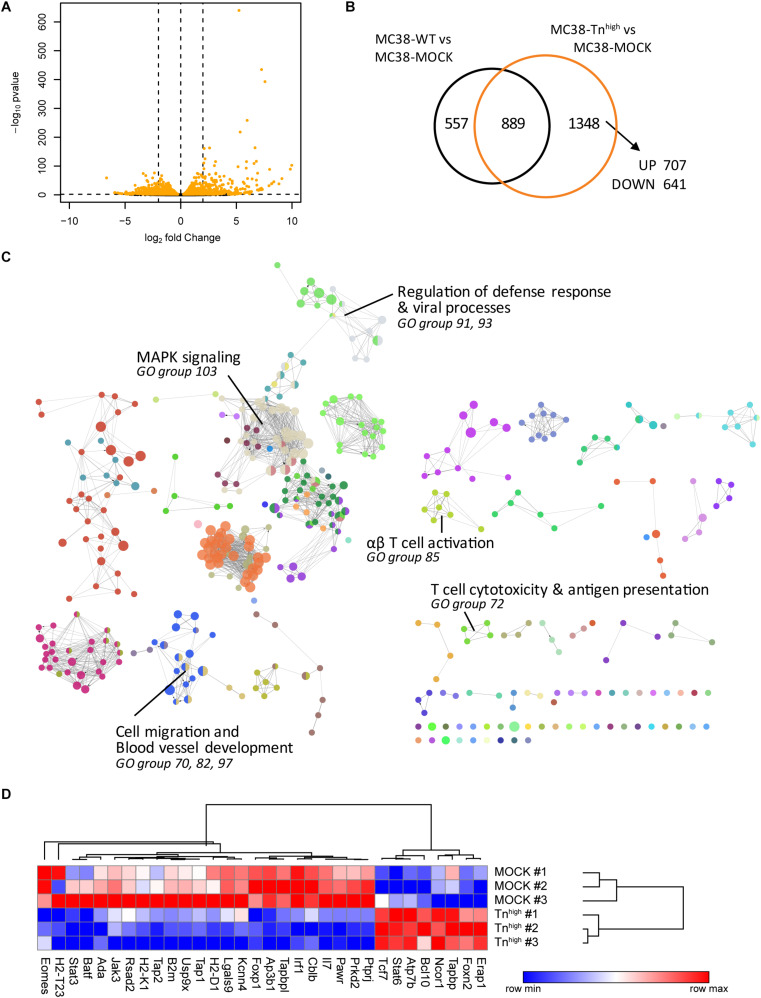
Knockout of *C1galt1c1* in tumor cells is associated with a decrease in antigen presentation and cytotoxic T cell activation. **(A)** Gene expression analysis in MC38-Tn^high^ cells compared to MC38-MOCK cells. Depicted are the log_2_ fold change and FDR-adjusted *p*-values in a minus log 10 transformation. Differentially expressed genes (DEGs) (FDR < 0.05) are highlighted in orange. **(B)** Comparative analysis of DEGs in MC38-WT vs MC38-MOCK cells and MC38-Tn^high^ vs MC38-MOCK cells. **(C)** Significant enriched GO terms annotated to the 1348 DEGs in MC38-COSMC KO cells. **(D)** Expression profile (log2 normalized counts) of the genes involved in antigen presentation and T cell activation.

To investigate whether increased expression of truncated *O*-glycans leads to transcriptional changes in the glycoengineered MC38 cells, we performed RNA sequencing (RNAseq) analysis in MC38-WT, MC38-MOCK and MC38-Tn^high^ cells. Comparative analysis revealed that the expression of 1,348 genes was specifically affected in MC38-Tn^high^ cells, of which 707 genes were increased in expression and 641 genes were downregulated (Figures 2A,B and Supplementary Tables 1–3). The effect of increased expression of truncated *O*-glycans by tumor cells on specific biological processes was assessed by subjecting the 1348 DEGs in MC38-Tn^high^ cells to GO term enrichment analysis (Figure 2C and Supplementary Table 4). We found evidence for effects of truncated *O*-glycans on MAPK signaling (GO group 103), cell migration, and blood vessel development (GO group 70, 82, 97) (Figure 2C, Supplementary Figures 2, 3 and Table 4), which corroborates previous reports (10, 31, 32). GO term enrichment analysis, furthermore, suggested an effect of truncated *O*-glycan expression on immune-related genes, involved in antigen processing and presentation, defense against viral infections, αβ T cell responses, and cytotoxic T cell responses (GO group 72, 85, 91, 93) (Figure 2C, Table 1 and Supplementary Table 4). Most of the genes involved in antigen presentation and T cell activation were suppressed in MC38-Tn^high^ cells (Figure 2D and Supplementary Tables 3–5), including the expression of well-known genes such as *B2m*, *Tap1*, *Tap2*, *Tapbp*, *H2-T23*, *H2-D1*, as well as the expression of important transcription factors, such as *Irf1*, *Batf*, *Foxp1*, *Eomes*, *Stat3*. *Stat6* was one the prominent genes upregulated in our MC38Tn^high^ cells (Figure 2D). A reduction in the MHC loading machinery and MHC class I molecules in tumor cells is known to restrict immune cell infiltration and to hamper anti-tumor immunity through the blockade of CD8^+^ T cell responses (33). STAT molecules play diverse pro- and anti-tumorigenic roles in cancer (34), however, in CRC high STAT3 expression is positively correlated to a better overall survival (35). Active STAT6 signaling seems to promote Thelper 2 cytokine profiles in CRC and to confer a pro-metastatic and anti-apoptotic tumor phenotype (36). Based on our RNAseq analysis, we hypothesized that truncated *O*-glycans and Tn antigen may have an immunomodulatory role in CRC.

**TABLE 1:** GO terms related to immune pathways.

GO group	GO ID	GO term	Corrected term *P* value	Nr. genes	Genes
Group 72	GO: 0002484	Antigen processing and presentation of endogenous peptide antigen via MHC class I via ER pathway	0.00598	3	*H2-D1*, *H2-K1*, *Tap2*
	GO: 0002485	Antigen processing and presentation of endogenous peptide antigen via MHC class I via ER pathway, TAP-dependent	0.00598	3	*H2-D1*, *H2-K1*, *Tap2*
	GO: 0001916	Positive regulation of T cell mediated cytotoxicity	0.02237	5	*[B2m*, *H2-D1*, *H2-K1*, *H2-T23*, *Tap2]*
	GO: 0001914	Regulation of T cell mediated cytotoxicity	0.04160	5	*B2m*, *H2-D1*, *H2-K1*, *H2-T23*, *Tap2*
Group 85	GO: 0046632	Alpha-beta T cell differentiation	0.01682	13	*Ada*, *Ap3b1*, *Atp7b*, *Batf*, *Eomes*, *Foxp1*, *Irf1*, *Jak3*, *Ncor1*, *Rsad2*, *Stat3*, *Stat6*, *Tcf7*
	GO: 0046631	Alpha-beta T cell activation	0.01917	16	*Ada*, *Ap3b1*, *Atp7b*, *Batf*, *Cblb*, *Eomes*, *Foxp1*, *H2-T23*, *Irf1*, *Jak3*, *Lgals9*, *Ncor1*, *Rsad2*, *Stat3*, *Stat6*, *Tcf7*
	GO: 0002293	Alpha-beta T cell differentiation involved in immune response	0.04065	8	*Atp7b*, *Batf*, *Eomes*, *Foxp1*, *Irf1*, *Jak3*, *Stat3*, *Stat6*
	GO: 0043367	CD4-positive, alpha-beta T cell differentiation	0.04165	9	*Atp7b*, *Batf*, *Foxp1*, *Irf1*, *Jak3*, *Ncor1*, *Rsad2*, *Stat3*, *Stat6*
	GO: 0002287	Alpha-beta T cell activation involved in immune response	0.04293	8	*Atp7b*, *Batf*, *Eomes*, *Foxp1*, *Irf1*, *Jak3*, *Stat3*, *Stat6*
	GO: 0035710	CD4-positive, alpha-beta T cell activation	0.04398	10	*Atp7b*, *Batf*, *Foxp1*, *Irf1*, *Jak3*, *Lgals9*, *Ncor1*, *Rsad2*, *Stat3*, *Stat6*
	GO: 0002360	T cell lineage commitment	0.04679	5	*Batf*, *Foxn2*, *Il7*, *Stat3*, *Stat6*
Group 91	GO: 0051607	Defense response to virus	0.00005	32	*Apobec3*, *Bnip3l*, *Bst2*, *Ddx58*, *Ddx60*, *Dtx3l*, *Gbp3*, *Ifih1*, *Ifitm1*, *Igf2bp1*, *Irf1*, *Irf5*, *Isg15*, *Oas1a*, *Oas1c*, *Oas1g*, *Oas2*, *Oas3*, *Oasl1*, *Parp9*, *Riok3*, *Rsad2*, *Rtp4*, *Samhd1*, *Tbk1*, *Trim12a*, *Trim30a*, *Trim30d*, *Trim34a*, *Zbp1*, *Zc3hav1*, *Zmynd11*
	GO: 0060760	Positive regulation of response to cytokine stimulus	0.00398	9	*Casp1*, *Casp4*, *Ddx58*, *Ifih1*, *Igf2bp1*, *Irgm1*, *Parp14*, *Parp9*, *Zbp1*
	GO: 0002753	Cytoplasmic pattern recognition receptor signaling pathway	0.00480	8	*5730559C18Rik*, *Ddx58*, *Ddx60*, *Erbin*, *Ifih1*, *Peli3*, *Riok3*, *Zc3hav1*
	GO: 0032728	Positive regulation of interferon-beta production	0.01026	7	*Ddx58*, *Ifih1*, *Irf1*, *Polr3a*, *Riok3*, *Tbk1*, *Zc3hav1*
	GO: 0031349	Positive regulation of defense response	0.01345	30	*5730559C18Rik*, *Cd47*, *Cd6*, *Cx3cl1*, *Ddx58*, *Ddx60*, *Erbin*, *H2-T23*, *Ifih1*, *Igf2bp1*, *Irak1*, *Irf1*, *Irgm1*, *Lgals9*, *Optn*, *Parp9*, *Peli3*, *Prkce*, *Ptgs2*, *Riok3*, *Rsad2*, *Snca*, *Tbk1*, *Tgm2*, *Tnfrsf1a*, *Trim30a*, *Trpv4*, *Ulbp1*, *Zbp1*, *Zc3hav1*
	GO: 0032481	Positive regulation of type I interferon production	0.02214	8	*Ddx58*, *Ifih1*, *Irak1*, *Irf1*, *Polr3a*, *Riok3*, *Tbk1*, *Zc3hav1*
	GO: 0045089	Positive regulation of innate immune response	0.02310	21	*5730559C18Rik*, *Ddx58*, *Ddx60*, *Erbin*, *H2-T23*, *Ifih1*, *Igf2bp1*, *Irak1*, *Irf1*, *Irgm1*, *Lgals9*, *Parp9*, *Peli3*, *Prkce*, *Riok3*, *Rsad2*, *Tbk1*, *Trim30a*, *Ulbp1*, *Zbp1*, *Zc3hav1*
	GO: 0039528	Cytoplasmic pattern recognition receptor signaling pathway in response to virus	0.02926	5	*Ddx58*, *Ddx60*, *Ifih1*, *Riok3*, *Zc3hav1*
	GO: 0039530	MDA-5 signaling pathway	0.03022	3	*Ddx60*, *Ifih1*, *Riok3*
	GO: 0045088	Regulation of innate immune response	0.04004	23	*5730559C18Rik*, *Ddx58*, *Ddx60*, *Erbin*, *H2-T23*, *Ifih1*, *Igf2bp1*, *Irak1*, *Irf1*, *Irgm1*, *Lgals9*, *Parp14*, *Parp9*, *Peli3*, *Prkce*, *Riok3*, *Rsad2*, *Samhd1*, *Tbk1*, *Trim30a*, *Ulbp1*, *Zbp1*, *Zc3hav1*
Group 93	GO: 0051607	Defense response to virus	0.00005	32	*Apobec3*, *Bnip3l*, *Bst2*, *Ddx58*, *Ddx60*, *Dtx3l*, *Gbp3*, *Ifih1*, *Ifitm1*, *Igf2bp1*, *Irf1*, *Irf5*, *Isg15*, *Oas1a*, *Oas1c*, *Oas1g*, *Oas2*, *Oas3*, *Oasl1*, *Parp9*, *Riok3*, *Rsad2*, *Rtp4*, *Samhd1*, *Tbk1*, *Trim12a*, *Trim30a*, *Trim30d*, *Trim34a*, *Zbp1*, *Zc3hav1*, *Zmynd11*
	GO: 0048525	Negative regulation of viral process	0.00029	16	*Apobec3*, *Banf1*, *Bst2*, *Ifitm1*, *Isg15*, *Oas1a*, *Oas1g*, *Oas3*, *Oasl1*, *Rsad2*, *Srpk2*, *Tfap4*, *Trim14*, *Zc3hav1*, *Zfp36*, *Zfp639*
	GO: 0045071	Negative regulation of viral genome replication	0.00034	11	*Apobec3*, *Banf1*, *Bst2*, *Ifitm1*, *Isg15*, *Oas1g*, *Oas3*, *Oasl1*, *Rsad2*, *Srpk2*, *Zc3hav1*
	GO: 0045069	Regulation of viral genome replication	0.00094	14	*Apobec3*, *Banf1*, *Bst2*, *Fkbp10*, *Ifitm1*, *Isg15*, *Oas1g*, *Oas3*, *Oasl1*, *Pabpc1*, *Ppia*, *Rsad2*, *Srpk2*, *Zc3hav1*
	GO: 0019079	Viral genome replication	0.00231	15	*Apobec3*, *Banf1*, *Bst2*, *Fkbp10*, *Ifitm1*, *Isg15*, *Oas1g*, *Oas3*, *Oasl1*, *Pabpc1*, *Ppia*, *Rsad2*, *Smarcb1*, *Srpk2*, *Zc3hav1*
	GO: 1903900	Regulation of viral life cycle	0.00476	17	*Apobec3*, *Banf1*, *Bst2*, *Fkbp10*, *Ifitm1*, *Isg15*, *Mvb12a*, *Oas1g*, *Oas3*, *Oasl1*, *Pabpc1*, *Pcx*, *Ppia*, *Rsad2*, *Srpk2*, *Trim30a*, *Zc3hav1*
	GO:1903901	Negative regulation of viral life cycle	0.00825	11	*Apobec3*, *Banf1*, *Bst2*, *Ifitm1*, *Isg15*, *Oas1g*, *Oas3*, *Oasl1*, *Rsad2*, *Srpk2*, *Zc3hav1*
	GO: 0045089	Positive regulation of innate immune response	0.02310	21	*5730559C18Rik*, *Ddx58*, *Ddx60*, *Erbin*, *H2-T23*, *Ifih1*, *Igf2bp1*, *Irak1*, *Irf1*, *Irgm1*, *Lgals9*, *Parp9*, *Peli3*, *Prkce*, *Riok3*, *Rsad2*, *Tbk1*, *Trim30a*, *Ulbp1*, *Zbp1*, *Zc3hav1*
	GO: 0045088	Regulation of innate immune response	0.04004	23	*5730559C18Rik*, *Ddx58*, *Ddx60*, *Erbin*, *H2-T23*, *Ifih1*, *Igf2bp1*, *Irak1*, *Irf1*, *Irgm1*, *Lgals9*, *Parp14*, *Parp9*, *Peli3*, *Prkce*, *Riok3*, *Rsad2*, *Samhd1*, *Tbk1*, *Trim30a*, *Ulbp1*, *Zbp1*, *Zc3hav1*
	GO: 0048524	Positive regulation of viral process	0.04398	10	*Fkbp10*, *Mvb12a*, *Pabpc1*, *Pcx*, *Ppia*, *Smarcb1*, *Srpk2*, *Tfap4*, *Trim30a*, *Zfp639*

*Genes were extracted from GO group 72, 85, 91, 93 (see also Supplementary Table 4).*

### Tn Antigen Drives Tumor Growth Specifically at Later Stages of Tumor Development

**FIGURE 3:**
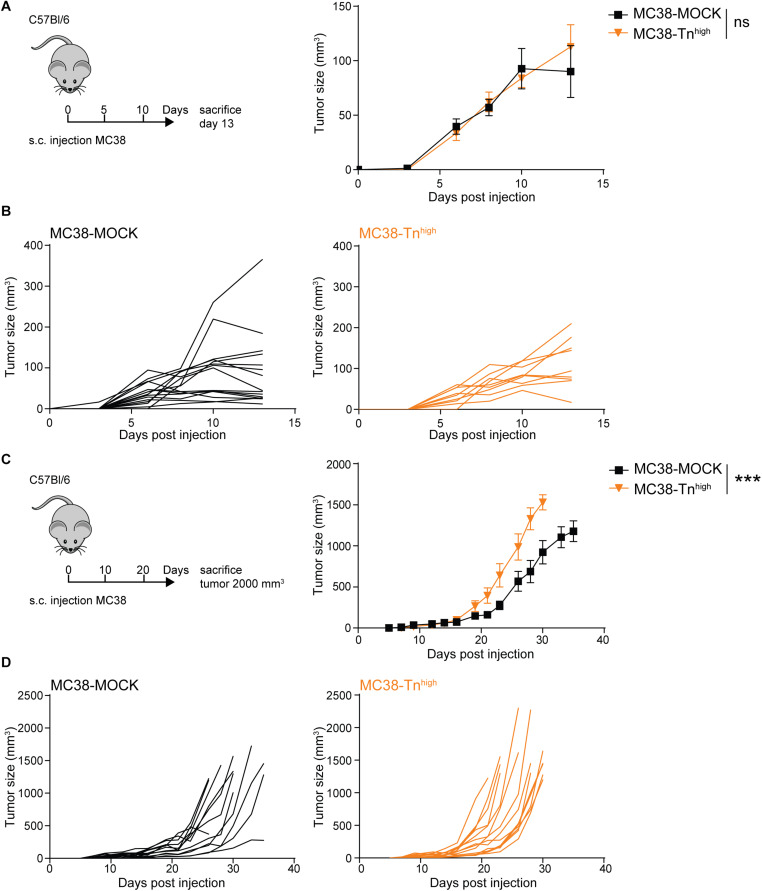
MC38-Tn^high^ tumors display enhanced tumor growth at later phases of tumor development. **(A–D)** MC38-MOCK or MC38-Tn^high^ cells were injected subcutaneously into C57Bl/6 mice and sacrificed at day 13 of tumor development (*n* > 10 mice/group) **(A)** or when tumor reached a size of 2000 mm^3^ (*n* = 14 mice/group) **(C)**. Tumor growth is depicted as mean tumor size **(A,C)** or individual tumor growth curves **(B,D)**. Mean ± SD; ***p < 0.001; ns, not significant.

We first explored the impact of high Tn antigen expression on tumor growth through subcutaneous injection of the MC38-MOCK and MC38-Tn^high^ cell lines in C57Bl/6 mice. Interestingly, and in contrast to a sialic acid knockout of MC38 (37), the *in vivo* growth rates were similar between MC38-MOCK and MC38-Tn^high^ tumors at day 13 of tumor development (Figures 3A,B). However, from day 23 onward MC38-Tn^high^ tumors started to display significantly faster growth compared to the MC38-MOCK tumors (Figures 3C,D). No differences in tumor growth were observed between male and female mice (data not shown). Thus, high Tn antigen expression seems to drive tumor growth particularly at later stages of colorectal cancer development.

### MC38-Tn^high^ Tumors Are Characterized by Lower Immune Cell Infiltration and Higher Levels of Myeloid-Derived Suppressor Cells

**FIGURE 4:**
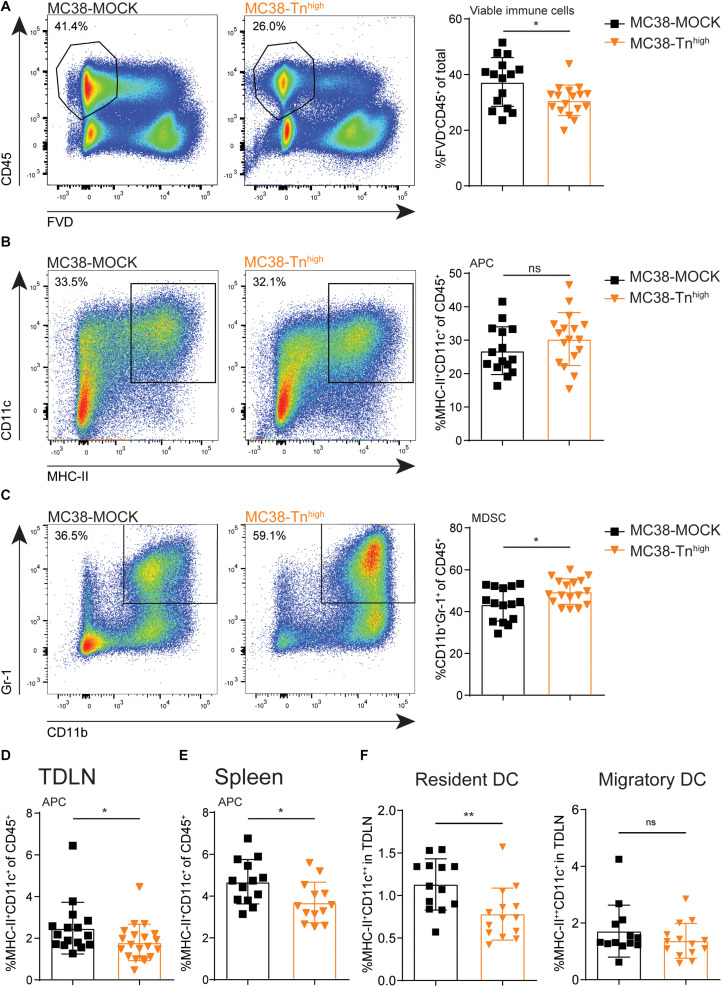
Characterization of myeloid immune cells in Tn^high^ tumors. **(A–C)** Flow cytometric analysis of viable immune cells (FVD^–^ CD45^+^) **(A)**, antigen presenting cells (APCs, MCH-II^+^CD11c^+^) **(B)** and myeloid derived suppressor cells (MDSCs, CD11b^+^Gr-1^+^) **(C)** at the tumor site. Tumors were harvested when they reached a size of 2000 mm^3^. **(D–F)** Flow cytometric analysis of APCs present in tumor draining lymph nodes (TDLN) **(D,F)** or spleen **(E)**. Data represents pooled data of *n* = 2 *in vivo* experiments with *n* > 5 and *n* > 8 mice/group **(A–D)** or one *in vivo* experiment with *n* = 13 mice/group **(E,F)**. Mean ± SD; *p < 0.05; **p < 0.01; FVD, fixable viability dye, ns, not significant.

Next, we examined the influence of tumor-associated Tn antigen on the immune cell composition within the tumor microenvironment. We inoculated mice with our MC38-MOCK and MC38-Tn^high^ and harvested the tumors when they reached a size of 2000 mm^3^. Strikingly, compared to MC38-MOCK tumors, MC38-Tn^high^ tumors displayed reduced infiltration of viable immune cells (FVD^–^CD45^+^) (Figure 4A). As carbohydrate binding lectin receptors are mainly expressed by antigen presenting cells (38), we first analyzed the myeloid immune cell compartment, including professional antigen presenting cells (APCs), such as dendritic cells (DCs) and macrophages, and myeloid-derived suppressor cells (MDSCs). APCs are known to internalize and present antigens to T cells, while MDSCs contribute to an immune suppressive tumor microenvironment (39). APC (MHC-II^+^CD11c^+^) frequencies were equal between MC38-Tn^high^ and MC38-MOCK tumors (Figure 4B), but were reduced in the tumor draining lymph node (TDLN, Figure 4D) and the spleen (Figure 4E). Interestingly, the reduced DC frequencies in the TDLN were confined to the lymph node-resident DC (CD11c^++^MHCII^+^) and not attributable to the migratory DC subset CD11c^+^MHCII^++^) (Figure 4F). Moreover, MDSC frequencies (CD11b^+^Gr-1^+^) (40) were significantly higher in MC38-Tn^high^ tumors compared to MC38-MOCK tumors (Figure 4C).

### MC38-Tn^high^ Tumors Display Reduced Cytotoxic CD8^+^ T Cell Frequencies

**FIGURE 5:**
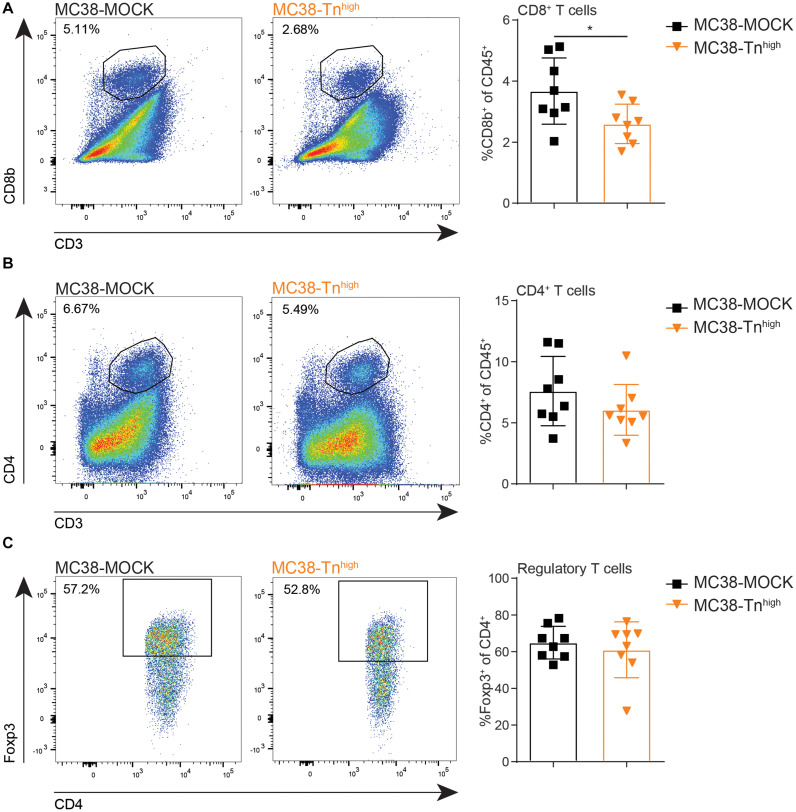
MC38-Tn^high^ tumors contain reduced numbers of cytotoxic CD8^+^ T cells. **(A–C)** Flow cytometric analysis of CD8^+^ T cells (FVD^–^ CD45^+^CD3^+^CD8b^+^) **(A)**, CD4^+^ T cells (FVD^–^ CD45^+^CD3^+^CD4^+^) **(B)** and regulatory T cells (Foxp3^+^ of CD45^+^CD3^+^CD4^+^) **(C)** at the tumor site. Tumors were analyzed when they reached a size of 2000 mm^3^. Data are representatives of *n* = 2 *in vivo* experiments. Mean ± SD; *p < 0.05; ns, not significant.

Resident DC in the TDLN are known to participate in the priming of tumor-specific CD8^+^ T cells through the capture of antigens from migratory DCs (41), suggesting that also adaptive T cell immunity could be affected in the MC38-Tn^high^ tumors. Moreover, high CD8^+^ T cell frequencies within the tumor microenvironment strongly correlate with a good prognosis in many cancer types (42), including CRC, supporting the substantial role of CD8^+^ T cells in combating cancer. Thus, we next analyzed the lymphoid compartment in our MC38-MOCK and MC38-Tn^high^ tumors. Indeed, compared to MC38-MOCK tumors, CD8^+^ T cell frequencies were decreased in MC38-Tn^high^ tumors (Figure 5A). Moreover, CD4^+^ T cell frequencies tended to be lower in MC38-Tn^high^ tumors, however, due to high variation within both groups, the differences observed were not significant (Figure 5B). Although reduced APC frequencies were found in TDLN of mice bearing MC38-Tn^high^ tumors (Figure 4D), T cell frequencies in TDLN were indistinguishable in the MC38-MOCK and MC38-Tn^high^ groups (Supplementary Figure 4). MDSCs are known to promote regulatory T cells expansion within the tumor microenvironment (43, 44), yet no increase in regulatory T cell frequencies was observed in MC38-Tn^high^ tumors (Figure 5C) or tumor-draining lymph nodes and spleen (Supplementary Figure 4).

We next analyzed the expression of immune checkpoint markers on the tumor-infiltrating CD8^+^ T cell population. Although equal percentages of PD-1^+^CD8^+^ T cells were found in MC38-MOCK and MC38-Tn^high^ tumors, the percentage of TIM-3^+^CD8^+^ T cells was significantly lower in the MC38-Tn^high^ tumors (Supplementary Figure 5A). Based on these data we cannot ascertain whether the CD8^+^ T cells in the MC38-Tn^high^ tumors are less exhausted or less activated, especially as the percentage of IFNγ-producing cells was not different upon *in vitro* PMA/Ionmycin restimulation of tumor-infiltrating lymphocytes from MC38-MOCK and MC38-Tn^high^ tumors (Supplementary Figure 5B).

In conclusion, MC38-Tn^high^ tumors displayed increased tumor growth at later stages of tumor development, which was characterized by enhanced levels of MDSCs and decreased levels of CD8^+^ T cell infiltration. Together these results suggest that MC38-Tn^high^ tumors are able to modulate the immune landscape within the tumor microenvironment, which might contribute to the enhanced tumor growth *in vivo*.

## Discussion

An aberrant glycosylation profile, including high Tn antigen expression, is a key feature of epithelial cancer types. Tumor-associated Tn antigen is found in 81–95% of CRC cases, independent of the histological subtype and differentiation status of the tumor, while it is not expressed by the healthy gut mucosa (10, 45, 46). Its expression has been correlated to bad prognosis and increased metastasis (5, 6), however, the effect of Tn antigen on the anti-tumor immune response has not been investigated in detail. In this study, we explored in a colorectal cancer mouse model how Tn antigen influences tumor growth and the immune cell composition in the tumor microenvironment. We show that knockout of the *C1galt1c1*/*Cosmc* gene alters the cell-intrinsic properties of tumor cells, affecting genes involved in MAPK signaling, cell migration and angiogenesis and immune regulation (Figure 2). Our results corroborate the work by Radhakrishnan et al., who also observed a downregulation of immune-related genes in *COSMC* knockouts of human keratinocytes (12). Finally, we provide evidence that enhanced Tn antigen expression coincided with enhanced tumor growth, especially at later stages of tumor development (Figure 3) and an alteration of the immune landscape within the tumor microenvironment (Figures 4, 5).

Tn antigen is the initial step of the *O*-glycosylation pathway and it can be further elongated in three distinct ways; through the addition of α2-6 sialic acid forming sialyl-Tn (sTn) antigen, with a galactose (forming the T antigen, Galβ1-3GalNacα-Ser/Thr) or a *N*–acetylglucosamine (core 3). Core 3 *O*-glycan structures are essential for intestinal mucus barrier function (47) and are responsible for colonic disease resistance (48). Also, the glycosyltransferase responsible for core 3 synthesis is generally downregulated during CRC progression, which actually accelerates CRC development (48, 49). Together, this implies an antagonizing role of core 3 in tumor growth, hence it is not likely that core 3 is responsible for the observed increase in MC38-Tn^high^ tumor growth. Sialyl-Tn (sTn) antigen has been described to support tumor progression as well (50) and could thus be accountable for the enhanced tumor growth of MC38-Tn^high^ cells *in vivo*. However, flow cytometric analysis of α2-6 sialic acids or sTn expression, ruled out the presence of α2-6 sialic acids in any of the cell lines used (data not shown), indicating that *de novo* Tn antigen expression was not sialylated in our MC38-Tn^high^ cells. Nevertheless, whether the effects we observed are due to an increase in Tn antigen or to a loss of the T antigen structure or longer *O*-glycans remain to be defined. In literature, conflicting reports exist on the influence of Tn or T antigen on tumor development. Overexpression of Tn antigen has been shown to promote tumor growth in CRC (11, 51) and pancreatic cancer (13). Moreover, in some models the mere abrogation of T-synthase/COSMC is sufficient to induce oncogenic features and to augment tumorigenesis of otherwise healthy cells (12, 52). In contrast, enhanced T antigen expression also correlates to bad prognosis and oncogenesis in, for instance, breast (31, 53, 54) and head and neck cancer (55). Strikingly, although Du et al. observed reduced breast cancer growth upon *COSMC* disruption, they also noticed an inhibition of the MAPK pathway similar to our findings (31) (Supplementary Figure 3). Thus, both Tn antigen and T antigen affect tumor progression and discrepancies might be explained by the tumor (sub)type or cell line investigated. Thus, whether Tn and T antigen work synergistically or whether one dominates over the other, remains to be investigated and is most likely tumor type-dependent.

The prominent role of anti-tumor immunity in CRC is clearly illustrated by the “Immunoscore,” a validated prognostic classification of CRC tumors according to their intratumoral density of memory and cytotoxic CD8^+^ T cells, superior to the classical TNM staging (56). Indeed, we also observed a decreased infiltration of CD8^+^ T cells in our MC38-Tn^high^ tumors, highlighting the critical role that these cells play in CRC progression. Although very little is known on the *in vivo* immune response to Tn antigen-positive tumors, *in vitro* work has already provided some clues. We postulate that the Tn antigen-dependent immune effects might be mediated through the interaction with lectin receptors expressed on immune cells. One prime candidate in this respect would be the Macrophage Galactose-type Lectin (MGL, CD301). MGL is expressed by (tolerogenic) dendritic cells (DCs) and macrophages (57) and has a unique preference for tumor-associated Tn and sTn antigen (58–60), through which MGL is able to distinguish between tumor-associated carbohydrate antigens and healthy tissue (61). Upon ligand binding, MGL signaling converges with Toll-like receptor-induced pathways, which increases the secretion of the anti-inflammatory cytokine IL-10 by human DCs and promotes the differentiation of human Tr1 cells by these DCs (62, 63). Moreover, MGL-mediated engagement of CD45 on effector T cells inhibits T cell cytokine secretion and can even induce T cell death (64), suggesting that tumor-associated Tn antigen might dampen adaptive immune responses via the MGL receptor at multiple levels to support tumor growth. Indeed, MGL ligand expression has been associated with lower survival in late stage CRC (65) and poor survival and distant metastasis in cervical squamous cell and adenosquamous carcinoma (66). Nevertheless, we cannot rule out that T and sialylated T antigen may also have immunomodulatory properties within the tumor microenvironment. COSMC KO of human breast cancer cells actually sensitized tumor cells to natural killer or cytotoxic CD8^+^ T cell killing (67), while sialyl-T was shown to promote the differentiation of tumor-associated macrophages through the engagement of inhibitory sialic acid-binding receptor Siglec-9, respectively (68). Clearly, more work is needed to elucidate the individual roles of truncated tumor-associated *O*-glycans on the anti-tumor immune response.

Interestingly, the association with MGL ligand expression was solely observed in stage III CRC and not in stage II CRC patients, which fits with our findings that Tn antigen augments tumor growth mainly from day 23 onward. Our data thus also implies that Tn antigen supports tumor progression and dampens anti-tumor immunity specifically during later stages of tumor development. Nevertheless, CRC is a very heterogeneous disease, comprising of four consensus molecular subtypes with different biological characteristics (69) and as yet, it is unclear how aberrant tumor glycosylation receptor impacts each CRC subtype. Certain glyco-gene signatures may be able identify patients groups with poor prognosis (70); however, the high incidence of Tn antigen (up to 80% in CRC) suggests that glycosylation-based immune modulation might be subtype-independent and should thus be regarded as a novel immune checkpoint across the whole CRC spectrum (14).

## Data Availability Statement

The datasets presented in this study can be found in online repositories. The names of the repository/repositories and accession number(s) can be found below: Sequence Read Archive (SRA) Gene Expression Omnibus (accession number GSE143700).

## Ethics Statement

The animal study was reviewed and approved by VU University Animal Welfare Commission, VU University, Amsterdam, Netherlands.

## Author Contributions

LC designed and performed experiments, analyzed data, and wrote the manuscript. AB, AZ, and JH performed the experiments. AZ analyzed RNA sequencing data. LK performed subcutaneous cell injections and mouse experiments. TO’T performed cell sorting experiments. YK and SV conceived and coordinated the study. SV designed and supervised the study and wrote the manuscript. All authors contributed to reviewing and/or revising the manuscript.

## Conflict of Interest

The authors declare that the research was conducted in the absence of any commercial or financial relationships that could be construed as a potential conflict of interest.

## Footnotes

1 https://www.bioinformatics.Babraham.ac.uk/projects/fastqc/

2 http://www.R-project.org/

3 https://bioinfogp.cnb.csic.es/tools/venny/index.html

4 https://cytoscape.org/

5 http://apps.cytoscape.org/apps/cluego

6 https://software.broadinstitute.org/morpheus/
