# Supplementary material for: Tn Antigen Expression Contributes to an Immune Suppressive Microenvironment and Drives Tumor Growth in Colorectal Cancer
Source: Front Oncol. 2020 Aug 18;10:1622. doi: 10.3389/fonc.2020.01622 (PMC7461972; doi:10.3389/fonc.2020.01622)
Supplement: Supplementary file 1 [file Data_Sheet_1.PDF]

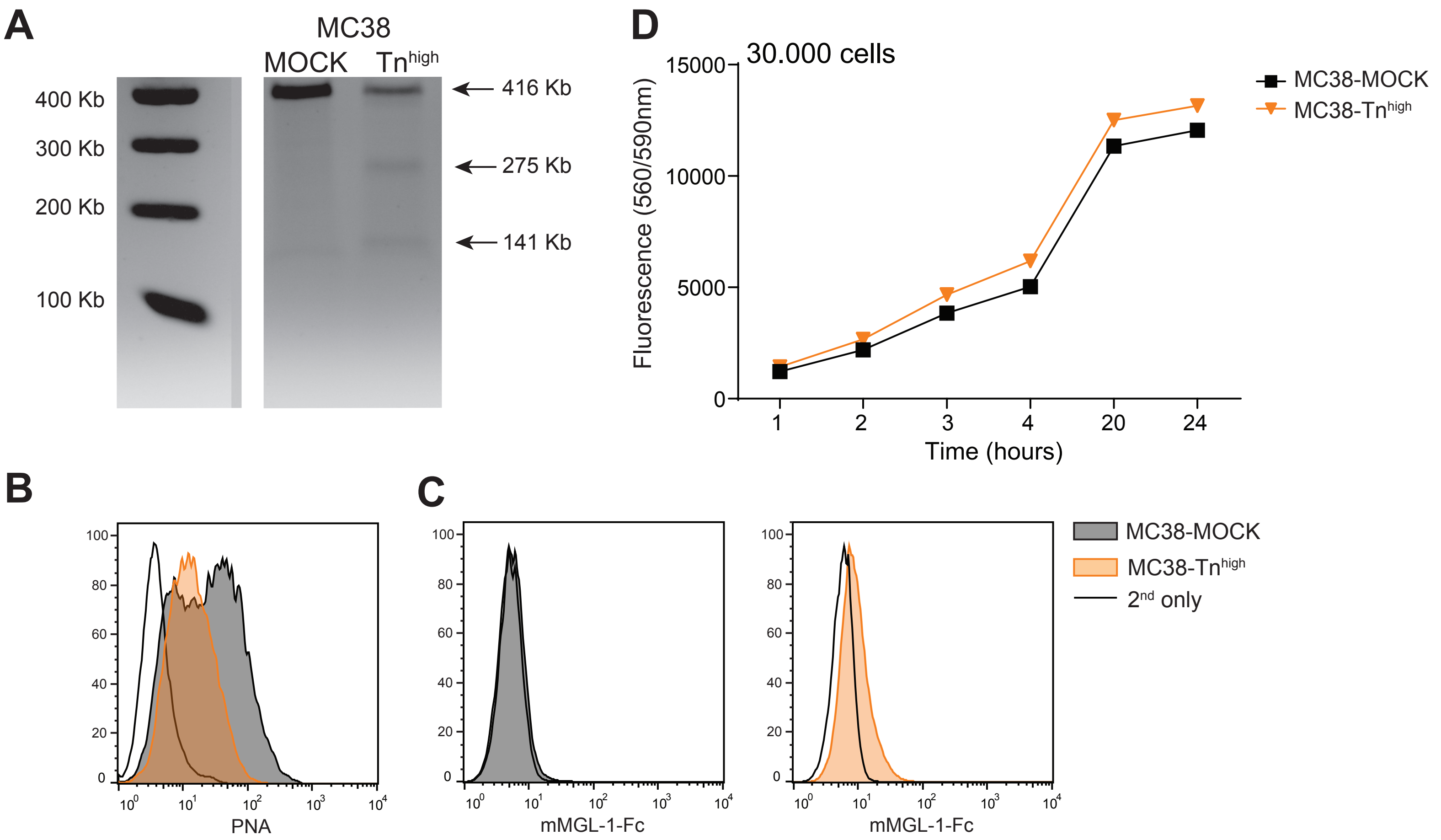

Supplementary Figure 1. Characterization and validation of the *C1galt1c1* knockout MC38-Tn^{high} cells. **(A)** The COSMC gene, C1galt1c1, was amplified with qPCR (product length 416 bp, top arrow) and tested for mutations using the Surveyor assay. The Surveyor nucleases recognizes and cleaves DNA mismatches. In the presence of a C1galt1c1 mutation the qPCR product is cleaved into two fragments of 275 bp (middle arrow) and 141 bp (bottom arrow). **(B-C)** Flow cytometric analysis of PNA (T antigen, B) and mouse macrophage galactose-type lectin 1 (mMGL-1-Fc) (C) binding to MC38-MOCK and MC38-Tn^{high} cells. **(D)** Metabolic cell activity assessed with the CellTiter-Blue® Cell Viability assay of MC38-MOCK and MC38-Tn^{high} cells after the first 24 hours of culture. No significant differences were found. Mean ± SD.

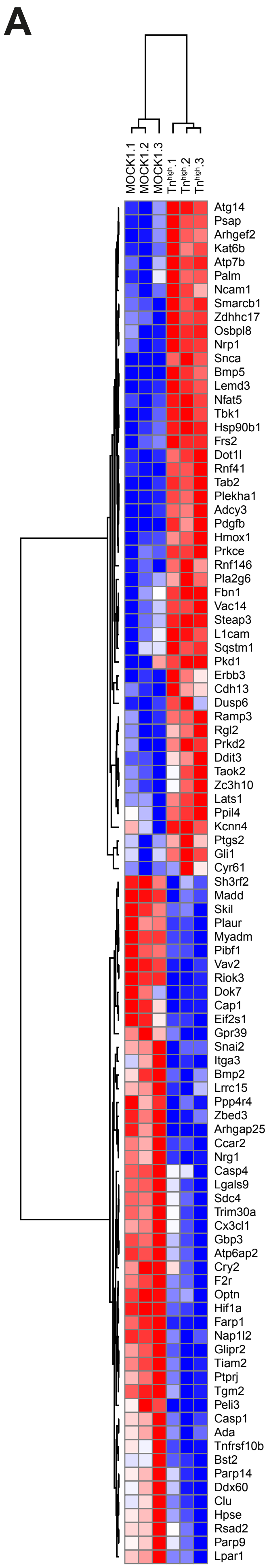

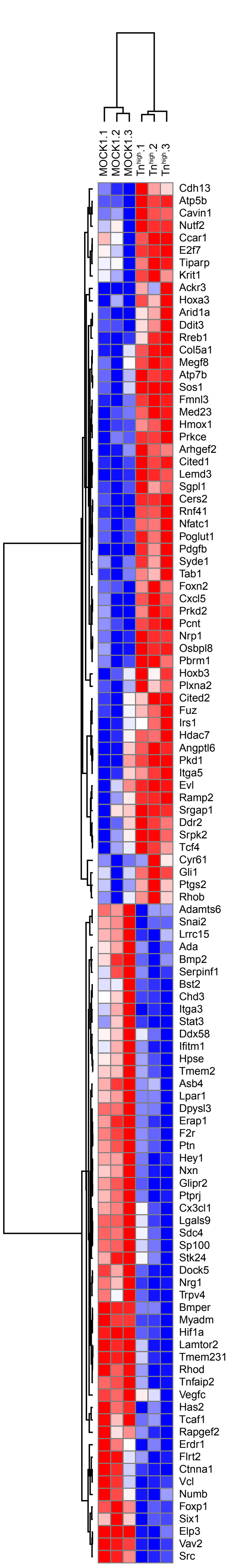

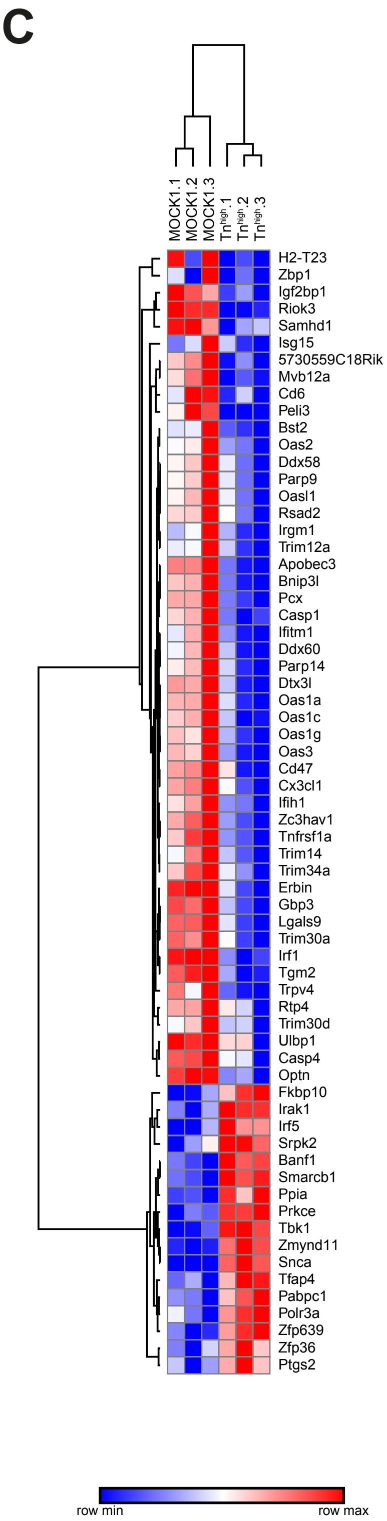

Supplementary Figure 2. Expression profile of the DEGs in COSMC KO involved in several biological processes. **(A)** Expression profile (log2 normalized counts) of the top 100 DEGs (based on FDR) associated with "MAPK signaling". In total, 219 genes were extracted from GO group 103 (**Figure 2C**; **Supplementary Table 4**). **(B)** DEGs associated with "Cell Migration" and "Blood vessel development". Genes were extracted from GO group 70, 82, and 97 (**Figure 2C**; **Supplementary Table 4**). **(C)** Expression profile (log2 normalized counts) of the DEGs in COSMC involved in the "Regulation of defense response" and "viral processes". Genes were extracted from GO group 91 and 93 (**Figure 2C**; **Supplementary Table 4**).

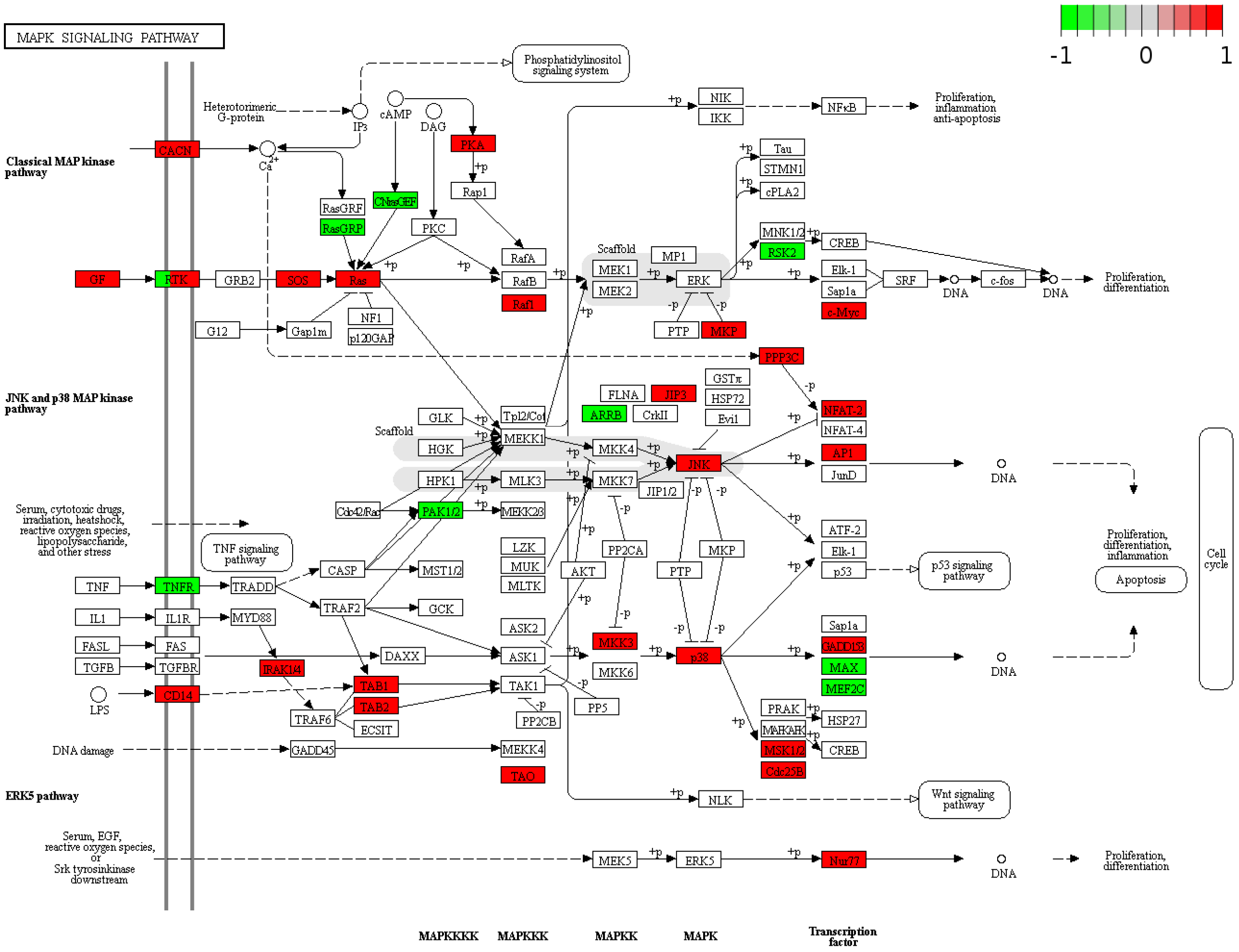

Supplementary Figure 3. DEGs involved in MAPK signaling pathway. The DEGs in MC38-Tn^{high} were visualized in the KEGG graph MAPK signaling pathway using Pathview (https://pathview.uncc.edu/).

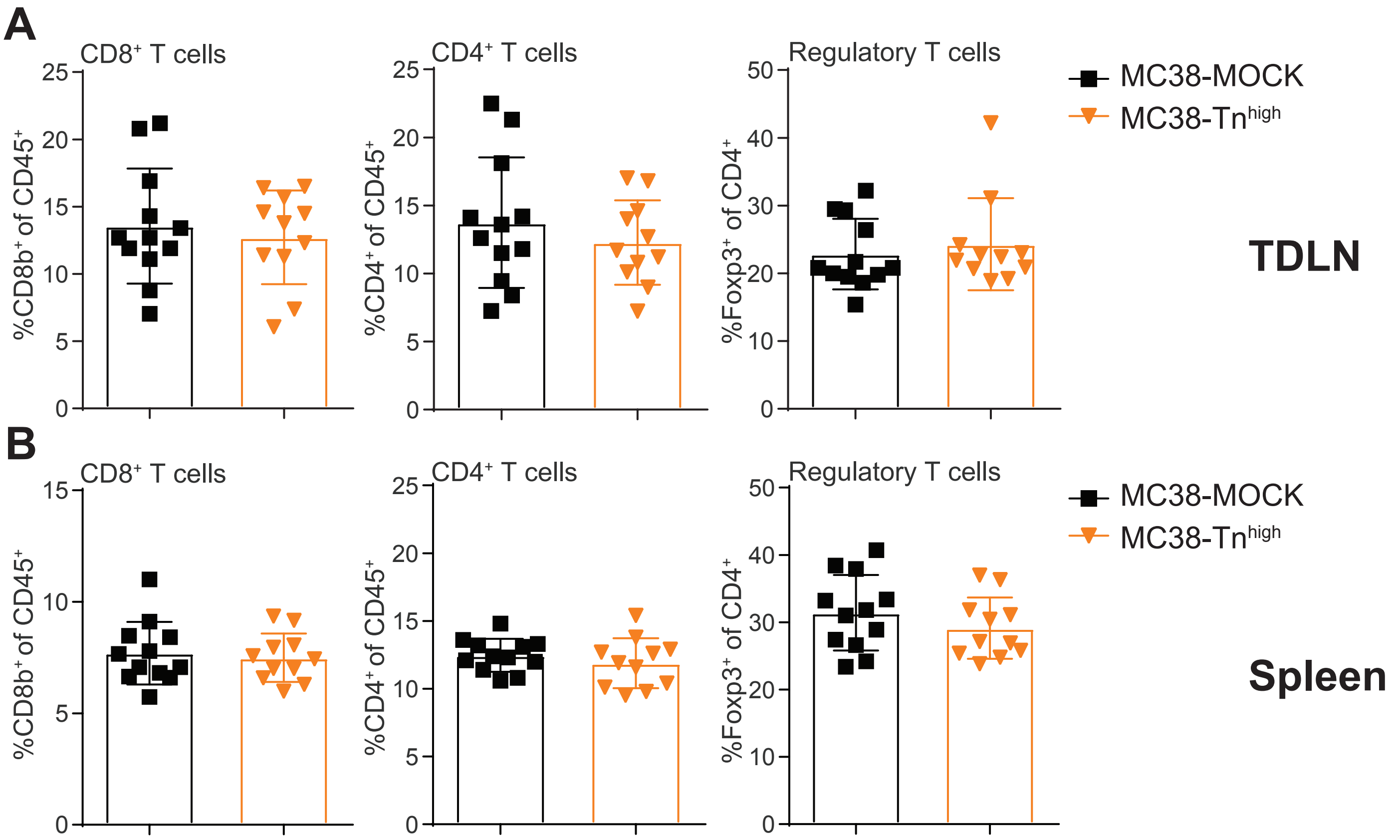

Supplementary Figure 4. T cells frequencies were unaffected in MC38-MOCK or MC38-Tn^{high} tumor-draining lymph nodes and spleen. **(A and B)** Flow cytometric analysis of $CD8^+$ T cells ($FVD^-CD45^+CD3^+CD8b^+$), $CD4^+$ T cells ($FVD^-CD45^+CD3^+CD4^+$) and regulatory T cells ($Foxp3^+$ of $CD45^+CD3^+CD4^+$) in the tumor-draining lymph nodes (TDLN) **(A)** or spleen **(B)**. T cell frequencies were analyzed when the tumor reached a size of 2000 mm^3. Data are representatives of n=2 *in vivo* experiments. Mean ± SD.

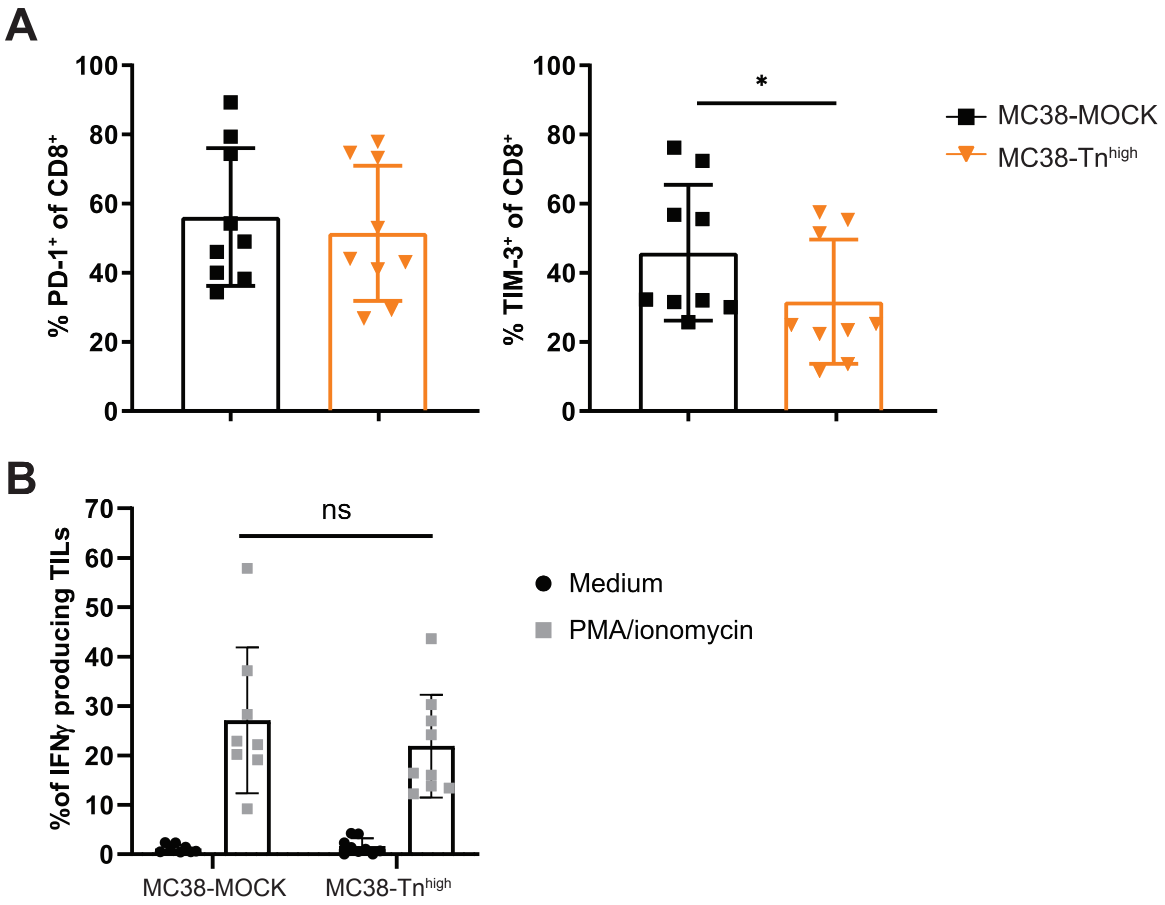

Supplementary Figure 5. MC38-Tn^{high} tumors contain less TIM-3 positive CD8^{+} T cells. **(A)** Flow cytometric analysis of PD-1 and TIM-3 expression on CD8^{+} T cells (FVD^{-}CD45^{+}CD3^{+}CD8b^{+}) in MC38-MOCK and MC38-Tn^{high} tumors. (**B**) Tumor-infiltrated lymphocytes were restimulated with PMA/ionomycin, stained for intracellular IFNγ and analyzed by flow cytometry. Data are representative of n=2 *in vivo* experiments. Mean ± SD.
